# Myocardial insufficiency is related to reduced subunit 4 content of cytochrome c oxidase

**DOI:** 10.1186/s13019-018-0785-7

**Published:** 2018-09-17

**Authors:** Sebastian Vogt, Volker Ruppert, Sabine Pankuweit, Jürgen P. J. Paletta, Annika Rhiel, Petra Weber, Marc Irqsusi, Pia Cybulski, Rabia Ramzan

**Affiliations:** 1 Cardiovascular Research Laboratories at the Biochemical Pharmacological Center, Philipps-University Marburg and Universitätsklinikum Gießen and Marburg GmbH, Marburg, Germany; 2 Heart Surgery, Philipps-University Marburg and Universitätsklinikum Gießen and Marburg GmbH, Marburg, Germany; 3 Department for Internal Medicine- Cardiology, Philipps-University Marburg and Universitätsklinikum Gießen and Marburg GmbH, Marburg, Germany; 4 Clinic for Orthopedics and Rheumatology, Philipps-University Marburg and Universitätsklinikum Gießen and Marburg GmbH, Marburg, Germany

**Keywords:** Cytochrome c oxidase, MT –CO_2_ and COX 4 expression, Myocardial insufficiency

## Abstract

**Background:**

Treatment of heart failure remains one of the most challenging task for intensive care medicine, cardiology and cardiac surgery. New options and better indicators are always required. Understanding the basic mechanisms underlying heart failure promote the development of adjusted therapy e.g. assist devices and monitoring of recovery. If cardiac failure is related to compromised cellular respiration of the heart, remains unclear. Myocardial respiration depends on Cytochrome c- Oxidase (CytOx) activity representing the rate limiting step for the mitochondrial respiratory chain. The enzymatic activity as well as mRNA expression of enzyme’s mitochondrial encoded catalytic subunit 2, nuclear encoded regulatory subunit 4 and protein contents were studied in biopsies of cardiac patients suffering from myocardial insufficiency and dilated cardiomyopathy (DCM).

**Methods:**

Fifty-four patients were enrolled in the study and underwent coronary angiography. Thirty male patients (mean age: 45 +/− 15 yrs.) had a reduced ejection fraction (EF) 35 ± 12% below 45% and a left ventricular end diastolic diameter (LVEDD) of 71 ± 10 mm bigger than 56 mm. They were diagnosed as having idiopathic dilated cardiomyopathy (DCM) without coronary heart disease and NYHA-class 3 and 4. Additionally, 24 male patients (mean age: 52 +/− 11 yrs.) after exclusion of secondary cardiomyopathies, coronary artery or valve disease, served as control (EF: 68 ± 7, LVEDD: 51 ± 7 mm). Total RNA was extracted from two biopsies of each person. Real-time PCR analysis was performed with specific primers followed by a melt curve analysis. Corresponding protein expression in the tissue was studied with immune-histochemistry while enzymatic activity was evaluated by spectroscopy.

**Results:**

Gene and protein expression analysis of patients showed a significant decrease of subunit 4 (1.1 vs. 0.6, *p* < 0.001; 7.7 ± 3.1% vs. 2.8 ± 1.4%, *p* < 0.0001) but no differences in subunit 2. Correlations were found between reduced subunit 2 expression, low EF (*r* = 0.766, *p* < 0.00045) and increased LVEDD (*r* = 0.492, *p* < 0.0068). In case of DCM less subunit 4 expression and reduced shortening fraction (*r* = 0.524, *p* < 0.017) was found, but enzymatic activity was higher (0.08 ± 0.06 vs. 0.26 ± 0.08 U/mg, *p* < 0.001) although myocardial oxygen consumption continued to the same extent.

**Conclusion:**

In case of myocardial insufficiency and DCM, decreased expression of COX 4 results in an impaired CytOx activity. Higher enzymatic activity but equal oxygen consumption contribute to the pathophysiology of the myocardial insufficiency and appears as an indicator of oxidative stress. This kind of dysregulation should be in the focus for the development of diagnostic and therapy procedures.

## Background

**Fig. 1:**
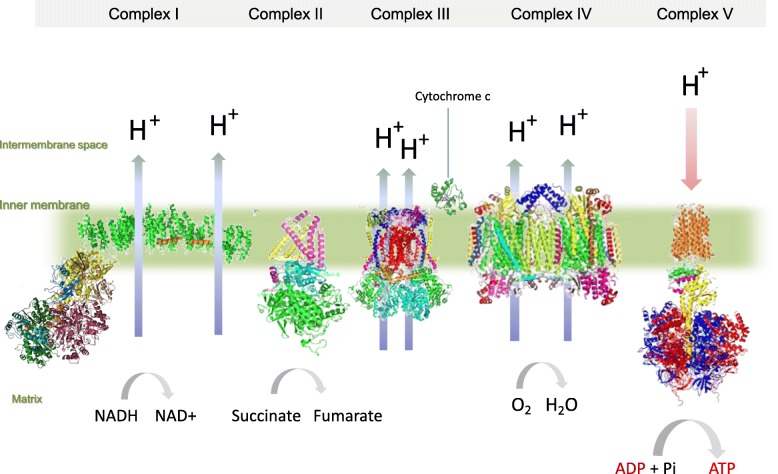
Mitochondrial electron transport chain (ETC) is composed of four multisubunit respiratory complexes and the carrier proteins that together perform mitochondrial respiration. Electrons are delivered from NADH and FADH_2_ to complex I and complex II, respectively, and subsequently passed through the electron transport chain to the final acceptor i.e. molecular oxygen to form water. This transfer of electrons through the ETC is associate with the translocation of protons at complexes I, III and IV across the mitochondrial membrane into the intermembrane space, thus trapping the energy in the form of electrochemical gradient., This energy stored in the gradient is used to perform oxidative phosphorylation i.e. formation of ATP by ATP synthase (complex V). Energy supply is closely related to the energy demand of the cell. Higher myocardial workload results in increased contractility spending more ATP and consuming more oxygen. Depending on the H^+^/e^−^ -stoichiometry and different efficiency of ATP synthesis, two different states of respiration (relaxed/active) may be considered [1–3]. A schematic representation of the enzymes involved in mitochondrial respiration and oxidative phosphorylation. Models of protein crystal structures were taken from the Protein Data Bank (https://www.rcsb.org/). The corresponding PDB IDs’ for complex I to V were: 2FUG, 1YQ3, INTZ, 10CC and 101

Deterioration of myocardial contractility is an obvious indicator for reduced oxygen supply. In coronary heart disease, when arteriosclerotic plaque formation reduces blood flow, the reduced coronary blood supply results in ischemia and damage to the myocardium. Myocardial respiration depends on Cytochrome c- Oxidase (CytOx) activity. It represents the rate limiting step for the function of the mitochondrial respiratory chain, also known as electron transmission chain (ETC). If cardiac failure is related to the compromised cellular respiration of the heart, remains unclear. But contractility requires abundant supply of adenosine triphosphate (ATP), and this kind of “energy currency” is produced in mitochondria (see Fig. 1) where oxygen consumption for water production at Cytochrome c- Oxidase (E.C. 1.9.3.1.) is a rate-limiting step. Decreased expression of COX 4 results in an impaired Cytochrome c oxidase activity [4, 5]. We hypothesize subsequent mitochondrial dysfunction associated with the formation of increased reactive oxygen species and inadequate maintenance of ATP levels when subunit 4 is reduced in the holoenzyme. This dysregulation is likely to contribute to the pathophysiology associated with myocardial insufficiency.

**Fig. 2:**
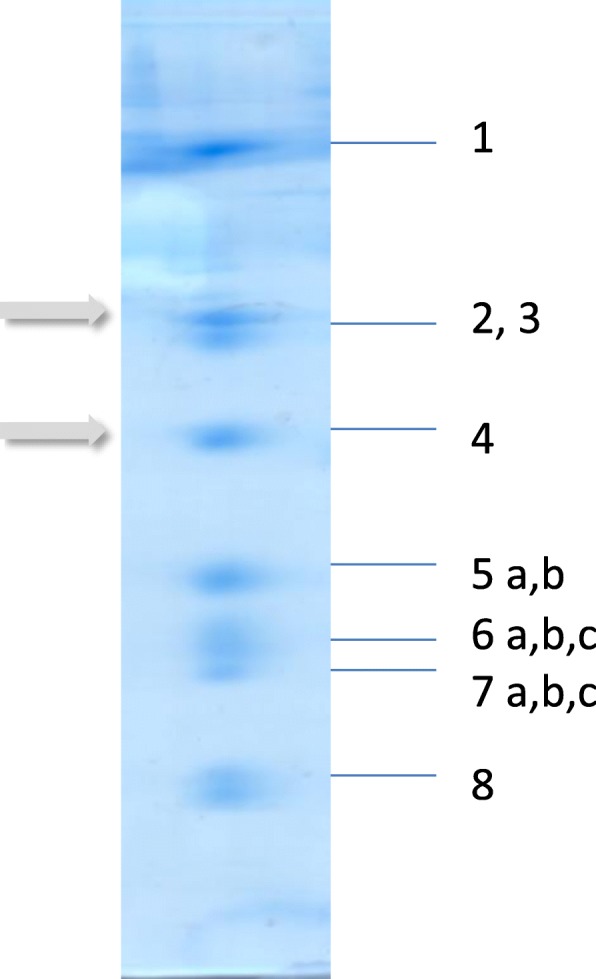
The subunit composition of the human ‘heart-type’ cytochrome c oxidase in 2D- gelelectrophoresis according to the *Kadenbach*- classification. Present study is focused on subunit 2 and 4 (arrows). CytOx from mammals and birds is composed of 13 subunits. The three catalytic subunits 1–3 are encoded by mitochondrial DNA, while 10 are nuclear-encoded. The latter ones are essentially involved in the regulation of oxygen consumption and proton translocation, since their removal or modification changes the activity and while their mutations may lead to mitochondrial diseases. Respiration is differently regulated in organs and species by expression of tissue-, developmental-, and species-specific isoforms for COX subunits 4, 6a, 6b, 7a, 7b, and 8, but the holoenzyme in mammals is always composed of 13 subunits [8]. Essential for the assembly and the oxygen consumption of the enzyme is the subunit 4 [9]. In the text, the subunits are termed in arabic numbers while mitochondrial enzyme complexes of the ETC are termed in latin numbers

The extent of ROS formation herein, determines the pathophysiological consequences. Ischemic damage and reperfusion injury of myocardium proceed with an *“Overspending ATP”* and finally metabolic break down. The CytOx (complex IV) is directly involved and has the “center stage” [4, 5]. This enzyme is considered to be the rate limiting step of electron transfer chain (ETC) [6, 7]. The holoenzyme in mammals is always composed of 13 subunits [8] (Fig. 2) where subunit 4 is essential for the assembly and oxygen consumption of the enzyme [9].

Interestingly, Buchwald et al. [10] found in hearts of transplant recipients with dilated cardiomyopathy (DCM) reduced cytochrome content as well as decreased complex IV activity. The group claimed no differences in subunit composition, although only gel- electrophoresis was used and harvested hearts represented profound end stage status of terminal cardiac insufficiency. Opposite data were presented by Grossmann and co-workers [11] where they found in homozygous and heterozygous Cox7a1 knockout mice, although viable, the reduced enzymatic activity and development of DCM at 6 weeks of age. Surprisingly, the cardiomyopathy improves and stabilizes by 6 months of age. Cox7a1 knockout mice incorporated more of the “liver-type” isoform Cox7a2 into cardiac type of the enzyme.

Mitochondrial mtDNA mutations are known to be involved in the pathogenesis of DCM [12, 13]. Point mutations are found more frequently. Mutations involving the evolutionary conserved residues of CytOx subunit 1 and NADH dehydrogenase 5 have been identified [14]. Additionally, further mutations were located in highly conserved domains of the gene coding for the CytOx [15, 16]. After cardiac transplantation, Arbustini and collaborators [14] found in explanted recipients’ hearts suffering from DCM, significant lower CytOx- activity related to NADH dehydrogenase and succinic dehydrogenase activities. They correlated pathological mtDNA mutations to CytOx- deficiency and abnormal mitochondrial ultrastructure.

The prognosis of DCM is poor because of its progression to myocardial insufficiency. Progression results either in cardiac death or prolonged support with inotropic medication, implantation of left ventricular assist devices, or heart transplantation, respectively. Effective, early indicators are required for diagnosing heart failure and commencing justified interventions in due time. Diversely, a good prognostic parameter to start the weaning process from cardiac assist devices under intensive medical care are needed as well.

Reflecting the need of such new parameters, the present study addresses mitochondrial pathophysiology of myocardial respiration in case of myocardial insufficiency. The study addresses the question, if reduced transcription and protein translation rates of subunit 4 represents a good indicator for compromised myocardial oxygen consumption and utilization for ATP production. The mismatch between mitochondrial- and nuclear coded subunits of CytOx indicates compromised cardiac dysfunction. Subunit 2 binds to one of the copper ions (CuA) whilst subunit 1 is likely to bind the two hemes (a and a_3_) and the other redox-active copper (CuB). Two cysteine and two histidine residues of subunit 2 are the likely ligands of CuA, forming a centre for electron transportation [17, 18]. Subunit 4 is known to be essential for the assembly and respiratory function of the enzyme complex [19, 20]. Accordingly, MT –CO_2_ /COX 4 gene expression, CytOx subunit 2 and 4 protein content and enzymatic activity of the holoenzyme in biopsies of DCM patients were determined. The extent of myocardial insufficiency in patients was detected by heart catheterization and LV angiography. Hemodynamic data were correlated to the biochemical findings of CytOx subunit 2 and 4 measurements.

## Methods

### Patients and dichotomization of the group

Patients with suspected inflammatory heart disease were referred to our hospital. All patients underwent heart catheterization. After exclusion of coronary artery disease, arterial hypertension and cardiac valve disease, up to 9 left ventricular (LV) endomyocardial specimens were obtained with a flexible Cordis bioptome from the free wall of the left ventricle, subsequently. All biopsies were snap-frozen and conserved in liquid nitrogen and stored at − 80 °C for maximum 2 months. The ESC-classification of cardiomyopathies of 2007 as well as the WHF-classification for the exclusion of inflammation and viral persistence was applied for the diagnosis of idiopathic dilated cardiomyopathy [21, 22]. Thirty patients (mean age: 45 +/− 15 yrs.) had reduced LV ejection fraction (EF: 35 ± 12%) below 45% and an enlarged left ventricular enddiastolic diameter bigger than 56 mm (LVEDD: 71 ± 10 mm). They were considered as myocardial insufficient and qualified for further analysis (Table 1). Furthermore, all of these patients were classified as having non-inflammatory, non-familial and non-viral forms of dilated cardiomyopathy by clinical work-up and in addition by immunohistochemical and molecular biological work-up of in total 5 endomyocardial biopsies. Twenty-four male patients (mean age: 52 +/− 11 yrs.) after final exclusion of inflammatory cardiomyopathy/ myocarditis or viral heart disease were also included in the investigation. They had improved hemodynamic data and served as a control group.

**Table 1:** Patients’ characteristics of both groups in comparison: Patients with suspected inflammatory heart disease were referred to our hospital based on recent onset of cardiac arrhythmias, undefined electrocardiogram changes, reduced exercise, tolerance or atypical chest pain. Coronary angiography was performed and endomyocardial specimens (*n* = 9) were obtained with a flexible Cordis bioptome from the free wall of the left ventricle*.* Patients with an EF < 45% and a LVEDD > 56 mm were considered having DCM disease. Medical treatment included Angiotensin-converting-enzyme-inhibitors 12/30 (40%), beta-blockers in 10/30 (33%) cases, diuretics in 10/30 (33%) cases, Angiotensin II receptor blockers in 27/30 (90%) cases. Anticoagulation with warfarin was made in 8/30 (27%) cases

	Age [y]	BMI	Nicotine	Diabetes	EF [%]	LVEDD [mm]	SF [%]
Controls (*n* = 24)	52 ± 11	28 ± 3	3/21 (12%)	2/21 (8%)	68 ± 7	51 ± 7	33 ± 12
DCM (*n* = 30)	45 ± 15	28 ± 5	5/18 (17%)	2/18 (7%)	35 ± 12	71 ± 10	20 ± 8

The endomyocardial biopsies used were the leftover samples from patients with suspected inflammatory disease after complete diagnostic work up. All patients gave their informed consent to use leftover samples for scientific purposes (Permission Ethics commission Az 53/18).

### mRNA expression analysis

Total RNA was extracted from the two pooled endomyocardial biopsies by QIAGEN™ RNeasy Kit according to the manufacturers’ instructions, including a DNase digestion (Serva™). The mRNA of the COX subunits 2 and 4 from heart tissue were amplified with specific primer pairs by one step real-time PCR using an iCycler (BioRad™). Polymerase chain reaction was carried out according to the manufacturers recommendations in a total volume of 25 μl containing 2.5 μl total RNA from endomyocardial biopsies as well as 7.5 μM cytochrome c oxidase 2, cytochrome c oxidase 4 or GAPDH (housekeeping gene), specific forward and reverse Primer and 2.5 μl SYBR Green. Primer sequences for GAPDH were, forward 5′-gAA ggT gAA ggT Cgg AgT C-3′ and reverse 5′- gAA gAT ggT gAT ggg ATT TC -3′; for MT- CO_2_ subunit-II, forward 5′- AgA CgC TAC TTC CCC TAT CA -3′ and reverse 5′- ggT CgT gTA gCg gTg AAA gT − 3′ and for CytOx subunit-4 were, forward 5′- gTA CGA gCT CAT gAA AgT gTT g − 3′ and reverse 5′- ACA TAg TgC TTC TgC CAC ATg A − 3′.

The protocols for “One Step Real-time PCR” used were as follows: reverse transcription 50 °C 30 min, denaturation (95 °C for 15 min), amplification repeated 40 times (95 °C for 45 s), annealing temperature (60 °C for 45 s), extension temperature (72 °C for 45 s). A melting curve analysis was run after final amplification period via a temperature gradient from 55 to 94 °C in 0.5 °C increment steps measuring fluorescence at each temperature for a period of 10 s. All reactions were carried out in at least 5 duplicates for every sample.

The relative expression of MT –CO_2_ and COX 4 transcripts were calculated as the ratio between the levels of transcript and GAPDH. Using the BioRad™ iQ iCycler system software, the threshold (Ct) at which the cycle numbers were measured, was adjusted to areas of exponential amplification of the traces. The ΔΔ-method was used to determine comparative expression level by applying the formula 2(-ΔCt target - ΔCt control) as described previously [23]. RNA content was compared in each biopsy by the distribution of GAPDH. All data were related to the GAPDH normalised ratio. The mean threshold cycles (CT) of all samples were similar (no significant differences *p* > 0.1) between these two groups). The analysis of intraindividual variance showed no difference of COX IV expression.

### Immunohistological examinations for protein expression

Immunohistochemical studies for the detection of the two subunits of the CytOx were performed on snap frozen biopsy specimens, conserved in liquid nitrogen. Biopsies were embedded in compound medium (Tissue-tek, Sakura Finetek, Torrance, U.S.A.) and serial sections of 6 μm were subsequently placed on poly-l-lysine–treated glass slides. Fixation was achieved by 100% ice-cold acetone. Endomyocardial biopsies of a subcohort of patients were subjected to staining with monoclonal antibodies against anti-human OxPhos Complex IV (MitoSciences, MitoSciences Inc., Eugene, OR 97403, U.S.A.) subunit 2 (clone 12C) and subunit 4 (clone 20E8C12) in a concentration of 1 μg/ml using a an avidin–biotin double sandwich technique (Vectastain Elite ABC Kit, Vector Laboratories, Burlingame, CA) in combination with a monoclonal antibody against the endothelial antigen EN4 (Sanbio, Am Uden, The Netherlands) to distinguish intramyocardial vessels or capillaries. Biopsy specimens were microscopically analyzed using a Leica DMRXE microscope. Histological analysis was carried out using a digital microscope (DM5000 Leica Microsystems, Bensheim, Germany) and QUIPS analysis software (Leica Microsystems, Bensheim, Germany). Evaluation was carried out by selecting 10 representative regions of interest (ROI) at 20-fold magnification. Relative stained area was calculated referring to the size of the biopsy.

### Myocardial oxygen consumption and enzymatic activity of CytOx

Biopsies were gently homogenized and suspended in KCl- buffer. Protein determination of myocardial suspensions were estimated by the BCA method with a NanoDrop™ instrument (NanoDrop 2000, Thermo Fisher Scientific™) using bovine serum albumin as standard. Concentration was fixed at 1 mg/ml.

Measurements of oxygen consumption were performed polarographically with Hansatech Oxygraph System (Hansatech Instruments Ltd., Norfolk, UK) using a Clark-type oxygen electrode as already described [24].

Enzymatic activity measurements were performed spectrophotometrically. The absorption of cytochrome c at 550 nm changes with its oxidation state. Cytochrome c was reduced with 0.1 M dithiothreitol solution and then reoxidized by the cytochrome c oxidase. The difference in extinction coefficients (Δε per mM) between reduced and oxidized cytochrome c is 21.84 at 550 nm. The oxidation of cytochrome c by cytochrome c oxidase appears as a biphasic reaction with a fast initial burst of activity followed by a slower reaction rate. Initial reaction rate is measured during the first 45 s of the reaction. The decrease in absorption at 550 nm at room temperature (25 °C) was monitored with a kinetic program: Reaction was started by the addition of 50 ml of Ferrocytochrome c (Merck KGaA, Darmstadt, Germany) as substrate. Absorption at 550 nm/minute was read immediately due to the rapid reaction rate of this enzyme. The final calculation of the sample activity in Units/ml according to: ΔA/min x Dilution × 1.1 ml (reaction volume)/ Volume of enzyme × 21.84 (Δε) and ΔA/min = A/minute – A/blank. The “Unit” is defined as 1 Unit oxidizes 1.0 mmole of ferrocytochrome c per minute at pH 7.0 (25 °C).

### Statistical analysis

Data were analyzed using the SigmaStat Advisory Statistics for scientists (SYSTAT®). For comparison of mRNA expression, CytOx levels and enzymatic activity in patients with DCM compared to controls, an unpaired, two-tailed t-test and a Mann-Whitney Rank Sum Test was used. A Fisher-test was used to demonstrate homogeneity of variances while in case of inhomogeneity, a Welch-test was performed. A *p*-value of less than or equal to 0.05 was considered significant. Linear regression analysis was also performed by SigmaStat™.

## Results

In this study, we have investigated the mRNA expression of MT –CO_2_ (mitochondrial encoded subunit) and COX 4 (nuclear encoded subunit) in endomyocardial biopsies (EMB) of patients with non- viral, non- inflammatory and non- familial idiopathic DCM and compared to controls. Hemodynamics of 54 patients were studied. Thirty patients were classified as DCM according to international criteria [21, 22]. In 24 individuals, neither infiltrating cells, cardiotropic virus, nor dilatation of the left or right ventricle and not even wall motion abnormalities were detected, although they had the clinical signs of infectious heart disease (recurrent fever, reported arrhythmia, dizziness etc.). These patients were considered as a control group (Table 1).

**Fig. 3:**
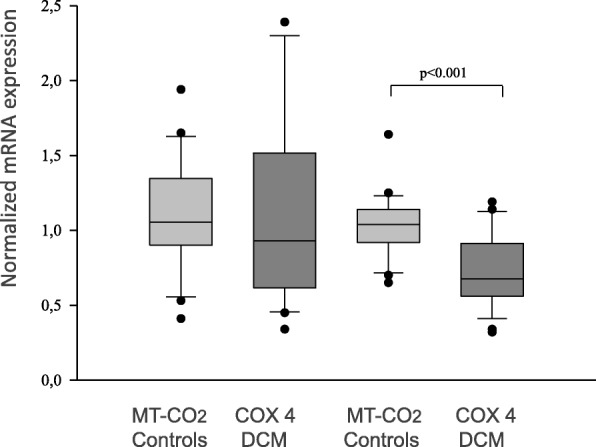
Relative mRNA expression of MT-CO_2_ and COX 4 in endomyocardial biopsies of patients with dilated cardiomyopathy (DCM) compared to controls

**Fig. 4:**
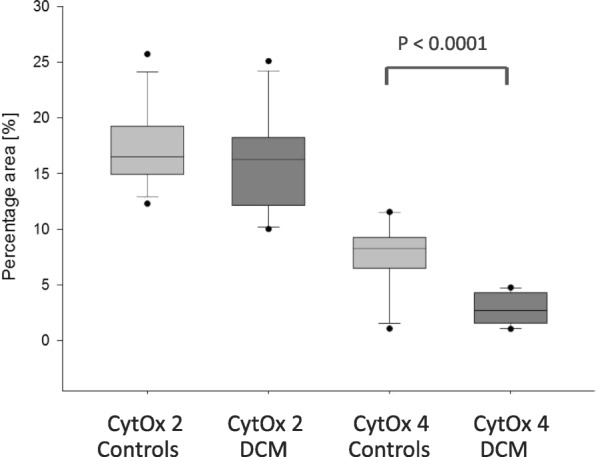
Immune histomorphometric detection of CytOx subunit 2 and 4 protein expressions in biopsies from DCM patients and controls. Evaluation shows clear reduction of CytOx subunit 4 protein expression in DCM tissue (*p* < 0.0001)

**Fig. 5:**
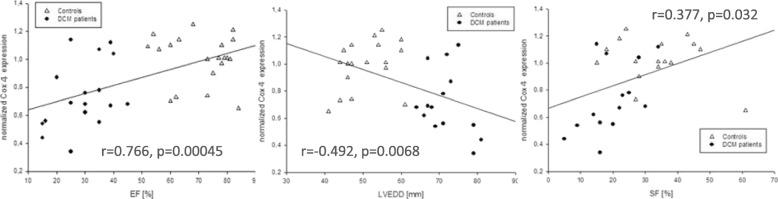
Correlations of normalized COX 4 expression vs. ejection fraction (EF) as well as left ventricular enddiastolic diameter (LVEDD) and shortening fraction (SF). Tests for significance of correlations are shown in the figure. In case of DCM less subunit 4 expression and reduced shortening fraction (*r* = 0.524, *p* < 0.017) was found

A significant decrease of COX 4 mRNA expression and protein content but no significant changes in correlating MT- CO_2_ measurements, were found (1.1 ± 0.2 vs. 0.6 ± 0.3, *p* < 0.001; 7.7 ± 3.1% vs. 2.8 ± 1.4%, *p* < 0.0001) (Figs. 3 and 4.) COX 4 mRNA expression data correlated with ejection fractions, EF (*r* = 0.766, *p* = 0.00045,) and LVEDD (*r* = − 0.492, *p* = 0.0068) as well as shortening fractions (SF; *r* = 0.377, *p* = 0.032) (Fig. 5). Otherwise, MT- CO_2_ did not correlate neither to EF and LVEDD nor shortening fractions (SF). The age of patients neither correlate to the MT- CO_2_ mRNA nor with COX 4 mRNA data. For this reason the found alterations were age- independent. Even in the group of DCM the reduced SF correlated to the reduced COX 4 expression (*r* = 0.52, *p* = 0.017).

**Fig. 6:**
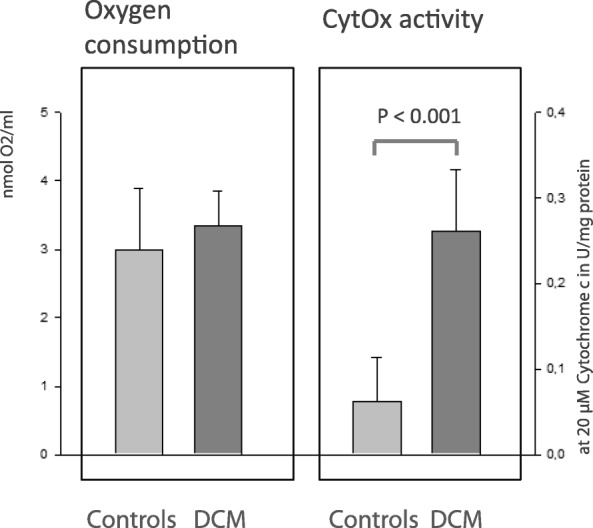
Protein content related to measurements of myocardial oxygen consumption in DCM group and control group (*p* = 0.643, n.s.) and simultaneous detection of CytOx activity in the presence of 20 μM reduced Cytochrome c (*p* < 0.001). Although the oxygen consumption rate is equal, the enzymatic activity is higher in the DCM group. The data indicates reduced efficiency in oxygen utilization as pathogenic factor for myocardial insufficiency and DCM, respectively

Interestingly, oxygen consumption testing provides no difference in both groups (3.1 ± 1.0 vs. 3.3 ± 0.6 nm/ml, *p* = 0.643, n.s.). However, enzymatic activity of CytOx in case of DCM was approximately 3fold increased (0.08 ± 0.06 vs. 0.26 ± 0.08 U/mg, *p* < 0.001, Fig. 6).

## Discussion

The question of myocardial oxygen supply, consumption and utilization remains to be one of the biggest problems in cardiovascular research [25]. Dilated Cardiomyopathy (DCM) is defined as a primary heart muscle disease with heart enlargement and impairment of the ventricular contractile function [26] and appears as a sufficient model to understand pathogenesis of myocardial insufficiency and cardiac failure, respectively. Mitochondrial dysfunction is a determinant of its pathogenesis [27]. In most cases, isolated CytOx- deficiencies are autosomal recessive disorders. Onset of the diseases starts at a very early age and is associated with fatal outcome (Leigh Syndrome and myopathies [28, 29]). Mutations in nuclear- encoded structural CytOx subunits have not been found in these phenotypes but still several assembly factors were affected. For example, SURF 1 (Leigh-syndrome), SCO2 (hypertrophic cardiopathy), SCO1 (hepatic failure) and COX 10 (sensorineural hearing loss, anemia, and hypertrophic cardiomyopathy) [16, 27, 30]. Saada and Coworkers [31] found a decreased mitochondrial thymidine kinase activity in association with mtDNA depletion responsible for certain kinds of myopathies. Reduced enzymatic activities of the electron transmission chain (ETC) in patients with dilated cardiomyopathy are found only in some cases [27]. The decrease of complex III activity was considered as a secondary phenomenon and not due to a mitochondrial defect for transcription and protein translation. In contrast, Hittel and coworkers found a differential expression of mitochondrial encoded genes in hibernation and specific increase of Complex IV- expression to prevent ETC alterations caused by cold and ischemia [32]. Evidence of a link between mutations in genes for respiratory chain components on one hand and human diseases on the other hand is reported with the emphasis on defects in respiratory complex IV and its assembly factors [27]. Early mutations in the mtDNA-encoded COX genes for CytOx subunits are relatively rare, but alterations in metabolism by thyroid hormones are known to influence the subunit composition at transcriptional and posttranscriptional level [33, 34]. Moreover increased mtDNA mutations are found with age in various human tissues as a result of oxidative stress. New aspect comes into consideration related to CytOx subunit composition in case of myocardial insufficiency and hypoxia with the emphasis on subunit 4 where the oxygen binding site of the holoenzyme is located [9].

Impairment of mitochondrial respiration and oxidative phosphorylation induces an increase in ROS production that causes mtDNA rearrangements, deletions and apoptosis [35]. A previous study found activities of complexes II and V of the ETC unchanged in dilated cardiomyopathy although cytochrome- containing complexes III and IV showed impaired activity, although subunit composition of CytOx remained the same as compared to the normal hearts in this study [10].

Opposite data addresses impaired cytochrome c oxidase- assembly in pathogenesis of myocardial insufficiency [36] and a heart-type cytochrome c oxidase subunit 7a1 was found to be associated with the development of DCM [37].

In our study, gene expression of mitochondrial encoded subunit 2 (MT-CO_2_) was throughout the same, but COX 4 was down-regulated and protein content of subunit 4 was also reduced in cases of myocardial insufficiency. This effect is age- independent. The complex IV in the ETC is involved in the electron transfer to dioxygen via subunit 4 that determines the subsequent respiratory activity of the tissue [6, 8, 9]. Exchange of bound ADP through ATP at a high-affinity binding site in the matrix domain of subunit 4 has a regulatory function for the whole enzymatic activity and for the final respiratory performance [38, 39]. Interestingly, in concern of previous findings [10] the enzymatic activity of CytOx in our estimations was found to be increased, but myocardial oxygen consumption had the equal amount in both groups. The data opens the question if down- regulation of subunit 4 is a consequence of hypoxia [40] or it represents the malfunctioning of the molecular subunit assembly among multiple factors [41] beside the uncoupling status of respiratory control [42]. Nonetheless, reduced subunit 4 appears as an indicator for myocardial insufficiency. Additional information in this regard has also been presented by the further analysis of subunit 4 isoforms (4i1 and 4i2) and their role in cardiac diseases [43].

New phase in heart surgery has begun. Testing indications and improving the outcome of patients in heart surgery require an understanding of molecular medicine and knowledge to be transferred at the bed- side level. In early studies, assessment of myocardial blood flow suggested myocardial ischemia as a reason for cardiac insufficiency and dilated cardiomyopathy [44]. Later studies corrected this idea [45]. Myocardial hypoxia has to be discussed as a matter of supply and demand and the different status of workload, respectively [46]. A third, new and an interesting fact with clinical relevance comes into consideration. Present study appears coherent with myocardial respiratory data from patients with left ventricular hypertrophy [47] and perhaps gives an explanation for a universal pathomechanism. The myocardial oxygen consumption is unchanged but efficiency is reduced. Different states of respiration determine efficiency of ATP synthesis and extent of ROS formation [1–3]. Quantity of ROS production results finally in myocardial damage and a progression of myocardial insufficiency, respectively.

## Conclusion

In this study, patients suspected for inflammatory heart disease and dilated cardiomyopathy (DCM) underwent heart catheterization. Thirty patients suffering from reduced LV ejection fraction below 45% and an enlarged left ventricular enddiastolic diameter bigger than 56 mm were considered to be myocardial insufficient. In biopsies of all patients the activity of Cytochrome c-oxidase (Complex IV of the mitochondrial respiratory chain) was measured. Furthermore, the subunit mRNA expression and protein content of the mitochondrial encoded catalytic subunit II (MT-CO2) and nuclear encoded regulatory subunit 4 (COX 4) were detected. Patients with myocardial insufficiency showed a significant decrease in COX 4 but not in MT-CO2. Correlations were found between COX 4 expression and EF% and LVEDD. Therefore, it is concluded that reduced COX 4 leads to impaired activity of Cytochrome c oxidase, subsequent reduction in mitochondiral respiration and hence myocardial insufficiency.

## Abbreviations

4i1 and 4i2: Subunit 4 isoforms of the Cytochrome c –Oxidase
COX 4: Gene expression of Cytochrome c Oxidase Subunit 4
CytOx: Cytochrome c –Oxidase
DCM: Dilated cardiomyopathy
EF: Ejection fraction
EMB: Endomyocardial biopsy
LV: Left ventricular
LVEDD: Left ventricular enddiastolic dimension
MT-CO_2_: Gene expression of Cytochrome c Oxidase Subunit 2
ROS: Reactive oxygen species
SF: Hortening fraction
